# Factorial Design and Optimization of Trimetallic CoNiFe-LDH/Graphene Composites for Enhanced Oxygen Evolution Reaction

**DOI:** 10.1021/acsaem.5c00483

**Published:** 2025-04-07

**Authors:** Daniele Alves, Gillian Collins, Marilia B. Dalla Benetta, Eithne Dempsey, Jae-Jin Shim, Raj Karthik, Carmel B. Breslin

**Affiliations:** † Department of Chemistry, Maynooth University, Maynooth, Co. Kildare W23 F2H6, Ireland; ‡ Kathleen Lonsdale Institute, Maynooth University, Maynooth, Co, Kildare W23 F2H6, Ireland; § School of Chemical Engineering, Yeungnam University, Gyeongsan 38541, Republic of Korea

**Keywords:** layered double hydroxide, graphene, electrocatalyst, factorial design, oxygen evolution reaction

## Abstract

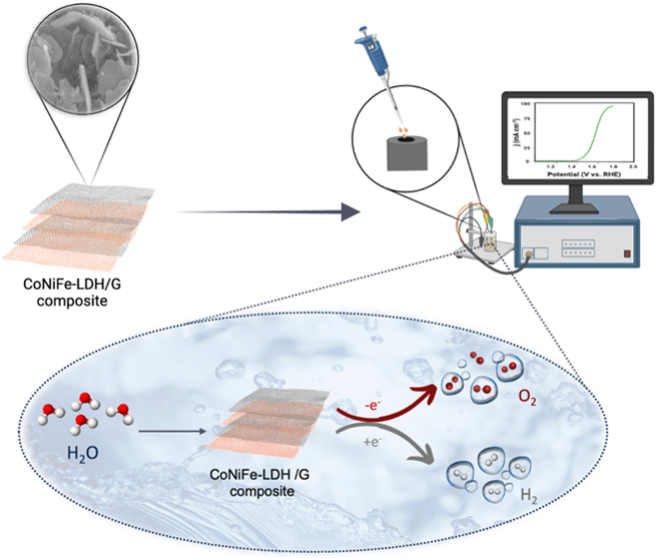

Layered double hydroxides (LDH) have exhibited promising applications as electrocatalysts in oxygen evolution reactions (OER). In this work, trimetallic LDHs (CoNiFe-LDH) were designed and grown on graphene (G) through a one-step hydrothermal approach to obtain a structure that promotes efficient charge transfer. A 2-level full-factorial design was utilized to evaluate the effects of varying the concentrations of Co (1.5, 3, and 4.5 mmol) and graphene (10, 30, and 50 mg) on the OER activity. The potential needed to deliver 10 mA cm^–2^ was chosen as the response parameter. The independent and dependent parameters were fitted to a linear model equation through ANOVA analysis. The computed *p*-values were below 0.05 signifying the statistical significance of the concentrations of cobalt and graphene and their interaction, suggesting a correlation with the OER activity. The OER experiments were conducted in triplicate using the Co_[3]_Ni_[3]_Fe_[3]_-LDH/G_[30]_ (central point) to estimate variability (0.58%). Comparative analysis showed that Co_[1.5]_Ni_[3]_Fe_[3]_-LDH/G_[10]_ achieved the lowest onset potential (1.54 V), potential at 10 mA cm^–2^ (1.58 V), and Tafel slope (58.4 mV dec^–1^), indicating that a low concentration of cobalt and graphene make an efficient electrocatalyst for OER. Furthermore, the optimized composite demonstrated favorable electronic properties, with a charge transfer resistance (R_CT_) of 188.1 Ω, and exhibited good stability, maintaining its catalytic activity with no significant loss over a 24-h period.

## 1 Introduction

For many decades, fossil fuels, such as gas, oil, and coal, have acted as the primary contributors to electricity generation.^1^ Nevertheless, the combustion of carbon-based fuels causes substantial emissions of greenhouse gases, notably carbon dioxide (CO_2_), thereby triggering climate change and adversely affecting human well-being and the environment.^2^ Consequently, there is an endeavor to curtail the utilization of fossil fuels and channel investments into renewable energy modalities, such as solar, wave, and wind power. Despite their environmental merits, these renewable sources are characterized by intermittency, making them incapable of ensuring a consistent energy supply.^3^ Consequently, increasing their proportion in the energy grid poses a big challenge. Thus, it is important to innovate and develop efficient and environmentally acceptable energy sources.

The oxygen evolution reaction (OER) constitutes a pivotal half-reaction within diverse renewable energy technologies. These include water electrolysis,^4^ metal-air batteries,^5^ and fuel cells.^6^ Essentially, it involves the conversion of water (H_2_O) into molecular oxygen (O_2_) through a four-electron transfer process. Despite its significance, the OER is intrinsically sluggish and energy-intensive, necessitating the advancement of electrocatalysts that are both efficient and stable to facilitate this reaction.^7^ Notably, noble metal-based materials, including IrO_2_ and RuO_2_, have shown superior OER performance under alkaline conditions.^8^ Nonetheless, their use is limited by high costs and limited reserves. Hence, the strategic design of novel electrode materials featuring low cost, high conductivity, and minimal overpotential assumes a crucial role in advancing electrocatalysis.

In recent years, layered double hydroxides (LDH) have gained recognition as possible candidates for the OER due to their cost-effectiveness, tunable composition, and favorable electrocatalytic properties.^9^ Ni-based LDHs, particularly NiFe-LDH, have been extensively studied and demonstrated as efficient OER electrocatalysts in alkaline environments.^10^ Due to its optimal adsorption energy for hydroxide anions, NiFe-LDH is recognized as a very good electrocatalyst for OER.^11^ However, its limited electrical conductivity hinders further enhancement of its OER catalytic activity.^12^ Meanwhile, Co-based LDHs, such as NiCo-LDH^13^ and CoFe-LDH^14^ have been proven to have excellent electrocatalytic activity.

In contrast to binary LDHs, the ternary LDHs, incorporating diverse transition elements can exhibit higher capacitance and contain more abundant active sites.^15^ Introducing a third metal ion in binary LDHs can alter the electronic structure and improve the conductivity, thereby increasing the density of active sites and facilitating more efficient electron transfer.^16^ Notably, Co-doping of NiFe-LDH, with vertical orientation, interconnections, and uniform distribution, has been shown to effectively enhance its electrocatalytic performance.^17^

Nevertheless, the utilization of LDH electrode materials is limited by their low conductivity and tendency for agglomeration. These challenges can be mitigated through the integration of LDH with carbon materials which exhibit good conductivity,^18^ and this includes graphene (G),^19^ carbon fibers,^20^ carbon nanotubes (CNT),^21^ and carbon cloth (CC).^22^ Graphene serves as an excellent substrate for catalyst immobilization in electrocatalysis, attributed to its remarkable electrical conductivity, large surface area, and impressive stability.^23^ This enhances the quantity of active sites, consequently improving electrochemical performance.^24^ Therefore, the combination of ternary LDHs, characterized by reversible redox activity, with conductive graphene is anticipated to offer a promising strategy for developing hybrid materials with superior OER activity, facilitated by the advantageous interplay between LDHs and graphene.^25^

Accordingly, in this work, the concentrations of cobalt and graphene, which serves as the carbon source, were optimized using a full-factorial design to formulate CoNiFe-LDH/G composites, aiming to enhance OER performance through an economical and efficient single-step hydrothermal reaction. This optimized composite was characterized and used as an electrocatalyst for the OER. Its performance was compared with the corresponding bimetallic components. This innovative approach employs statistical analysis to improve the efficiency of material preparation, leading to enhanced OER performance. Additionally, to the best of our knowledge, CoNiFe-LDH combined with graphene nanoplatelets has not previously been explored as an electrocatalyst for OER.

## 2 Experimental Section

### 2.1 Materials

The cobalt (Co(NO_3_)_2_·6H_2_O), nickel (Ni(NO_3_)_2_·6H_2_O) and iron salts (Fe(NO_3_)_3_·9H_2_O), urea (NH_2_CONH_2_), graphene nanoplatelets and RuO_2_ were all obtained from Sigma-Aldrich, UK. A glassy carbon electrode (GCE, 3 mm diameter) was used as a support for the CoNiFe-LDH/G composites.

### 2.2 Synthesis of Co_[m]_Ni_[3]_Fe_[3]_-LDH/G_[n]_ Composites

The Co_[m]_Ni_[3]_Fe_[3]_-LDH/G_[n]_ composites were synthesized using a hydrothermal method. The Ni and Fe content was maintained constant, while the amounts of graphene and Co were varied to give different molar ratios of Co to Ni and Fe (Co:Ni:Fe = 0.5:1:1 or 1:1:1 and 1.5:1:1). The Co and graphene levels were varied to investigate their combined effects on the OER activity. Experimentally, these composites were synthesized according to the procedures developed in the previous works,^14,26−28^ using a 2-level full factorial design, as illustrated in Table 1. The graphene nanoplates (10, 30, or 50 mg, Table 1) were dispersed in a 10 mL solution of deionized water and ethanol (equal volumes), and then sonicated for 20 min. A second solution containing Co(NO_3_)_2_ at different concentrations (1.5, 3, or 4.5 mmol, Table 1), combined with 3 mmol of Ni(NO_3_)_2_, and 3 mmol of Fe(NO_3_)_3_ in 20 mL of deionized water was prepared. Then, a third solution was obtained by dissolving 1.5 g of urea in 10 mL of deionized water. All three solutions were mixed, followed by stirring for 1 h to ensure complete dissolution. The resulting mixture was carefully transferred into a 100 mL Teflon-lined stainless-steel autoclave and maintained at 120 °C for 12 h. The Co_[m]_Ni_[3]_Fe_[3]_-LDH/G_[n]_ composites were washed several times with ethanol and deionized water, collected and then dried at 60 °C for 18 h. To verify the elemental composition, X-ray energy-dispersive spectroscopy (EDX) was conducted in triplicate for all composites, with the results summarized in Table 2. The elemental composition of the Co_[m]_Ni_[3]_Fe_[3]_-LDH/G_[n]_ composites (Table 2) aligns with the precursor concentrations (Table 1), reflecting controlled Co and graphene (%C) variation while maintaining constant Ni and Fe (standard deviation <0.8%). Additionally, to evaluate the influence of each component on the electrochemical performance and OER activity, a series of control materials with different compositions were synthesized using the same method, and named CoNiFe-LDH, NiFe-LDH/G, CoNi-LDH/G, and CoFe-LDH/G.

**Table 1:** Factors and Levels for the Optimization of the CoNiFe-LDH/G Composites

composites	1^st^ factor [Co]	2^nd^ factor [G]	[Co] mmol	[G] mg
Co_[1.5]_Ni_[3]_Fe_[3]_-LDH/G_[10]_	–1	–1	1.5	10
Co_[4.5]_Ni_[3]_Fe_[3]_-LDH/G_[10]_	+1	–1	4.5	10
Co_[1.5]_Ni_[3]_Fe_[3]_-LDH/G_[50]_	–1	+1	1.5	50
Co_[4.5]_Ni_[3]_Fe_[3]_-LDH/G_[50]_	+1	+1	4.5	50
Co_[3]_Ni_[3]_Fe_[3]_-LDH/G_[30]_	0	0	3	30

**Table 2:** Composition in % Atomic of the CoNiFe-LDH/G Composites

composites	Co % atomic^a^	Ni % atomic^a^	Fe % atomic^a^	C % atomic^a^	O% atomic^a^
Co_[1.5]_Ni_[3]_Fe_[3]_-LDH/G_[10]_	4.3 ± 0.5	5.9 ± 0.4	6.4 ± 0.5	38.6 ± 2.1	44.8 ± 2.7
Co_[4.5]_Ni_[3]_Fe_[3]_-LDH/G_[10]_	9.3 ± 0.9	5.6 ± 0.7	6.1 ± 0.6	37.5 ± 1.9	41.5 ± 2.5
Co_[1.5]_Ni_[3]_Fe_[3]_-LDH/G_[50]_	4.0 ± 0.5	5.8 ± 0.7	6.3 ± 0.6	48.1 ± 2.6	35.8 ± 2.2
Co_[4.5]_Ni_[3]_Fe_[3]_-LDH/G_[50]_	8.9 ± 0.7	5.6 ± 0.6	6.3 ± 0.8	47.4 ± 2.4	31.8 ± 2.1
Co_[3]_Ni_[3]_Fe_[3]_-LDH/G_[30]_	5.8 ± 0.6	5.7 ± 0.5	6.2 ± 0.7	42.8 ± 2.4	39.5 ± 2.6

a Mean ± standard deviation (*n* = 3).

### 2.3 Characterization

The optimized Co_[m]_Ni_[3]_Fe_[3]_-LDH/G_[n]_ and its graphene-free counterpart, Co_[m]_Ni_[3]_Fe_[3]_-LDH, were chosen for further investigation through structural characterization and electrochemical analysis. X-ray photoelectron spectroscopy (XPS) (Kratos AXIS ULTRA spectrometer) was conducted using monochromatic Al Kα radiation (1486.58 eV, 300 W, 20 mA, 15 kV) to determine surface element composition and valence state of the as-synthesized composite. The sample morphologies were examined via field emission scanning electron microscopy (FE-SEM, Hitachi S-4800), while the elemental composition was analyzed using energy-dispersive X-ray spectroscopy (EDX, Oxford Instrument INCAz-act ESX system). X-ray diffraction (XRD) analysis was conducted using a Powder X-ray PANalytical X’Pert-PRO MPD system to investigate the crystal structure of the samples. The measurements were performed with Cu Kα radiation (λ = 1.5406 Å) at an operating voltage of 40 kV, while the composites were also analyzed by Fourier transform infrared spectroscopy (FTIR, Nicolet iS50 FTIR spectrometer) in the range 4000–500 cm^–1^. The potential leaching of Co, Ni and Fe from the LDH composite was analyzed using inductively coupled plasma mass spectrometry (ICP-MS) (7900 ICP-MS, Agilent, Japan). The plasma was generated using argon gas (99.99% purity) with a 15 L min^–1^ plasma flow rate, while the auxiliary and nebulizer gases were supplied at 1 L min^–1^.

### 2.4 Electrochemical Measurements

The OER activity of the LDH electrocatalysts was studied at a polished and thoroughly cleaned GCE. The GCE was polished sequentially with 1 and 6 μm Akasol diamond suspensions on an Aka–Napel microcloth, followed by thorough rinsing with deionized water, sonication in deionized water and dried under an air flow. The LDH catalyst ink was formulated by mixing 3 mg of the LDH with 0.5 mL deionized water and 0.5 mL ethanol, followed by sonication for 10 min to ensure homogeneity. The resulting ink was applied to the GCE via drop casting, achieving an approximate catalyst loading of 84.9 μL cm^–2^.

The electrochemical measurements were carried out in 1 M KOH (pH 13.6) in a three-electrode cell, with the LDH-modified GCE, a silver/silver chloride (Ag/AgCl) reference, and a high surface area platinum wire counter electrode. Linear sweep voltammetry (LSV) was conducted at a scan rate of 5 mV s^–1^. All potentials were converted to the reversible hydrogen electrode (RHE) scale (*E*_RHE_ = *E*_Ag/AgCl_ + 0.197 V + 0.059 × pH) and were *iR*-corrected. Current densities were normalized to the geometric surface area of the GCE. Electrochemical impedance spectroscopy (EIS) measurements were performed over a frequency range of 1 × 10^6^ to 0.007 Hz at 1.58 V (RHE) using a 10 mV perturbation potential. The obtained impedance data were fitted to an equivalent circuit model to determine the charge transfer resistance. In addition, the stability of the selected trimetallic LDH, Co_[m]_Ni_[3]_Fe_[3]_-LDH/G_[n],_ was evaluated over a 24 h-period under a constant applied potential of 1.58 V (RHE). Following the 24 h polarization period, the EIS response of the LDH was measured and compared to the response of the freshly prepared LDH. Additionally, to assess the stability of the composite under conditions relevant to scaled-up electrochemical systems, which generally require higher current densities, a chronoamperometry experiment was conducted at a constant potential of 1.66 V (RHE), equivalent to 50 mA cm^–2^ for 72 h-period.

### 2.5 Statistical Analysis

Linear models were employed to analyze the OER response variable, incorporating replication as a factor with three levels. A linear response surface accounting for Co and G concentrations in the linear predictor for the mean was employed. The interaction effect of Co and G concentrations on the potential required to achieve 10 mA cm^–2^ was evaluated using F-tests. A contour plot was generated to illustrate the predicted OER value over a continuous 2D grid for varying concentrations of Co and G.

## 3 Results and Discussion

### 3.1 Alkaline Electrolyte Preparation and Evaluation

**Figure 1:**
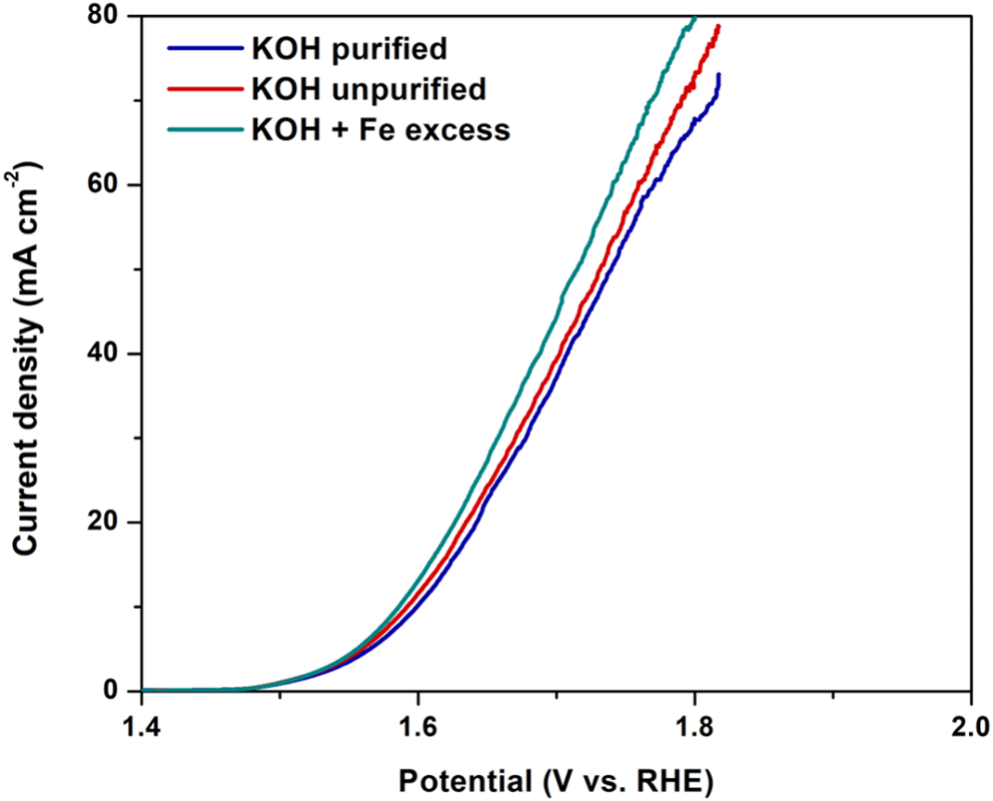
OER polarization curves using Co_[1.5]_Ni_[3]_Fe_[3]_-LDH/G_[10]_ in 1 M KOH solution, in purified, unpurified, and Fe-excess conditions.

Alkaline aqueous electrolytes, such as KOH and NaOH, are crucial for energy devices, including electrolyzers, fuel cells, supercapacitors, and batteries.^29^ Recent findings have shown that Fe impurities can notably impact the OER performance of Ni-based electrocatalysts.^30^ Therefore, addressing impurities is vital to assess alkaline electrolyte quality for objective evaluation and comparison of electrochemical energy systems. Based on this, CoNiFe-LDH/G was initially employed for OER in 1 M KOH solutions in unpurified, supplemented with excess Fe (20 ppm) and purified conditions to assess the effect of Fe ions on the electrocatalytic performance of the LDH composite. For the purification process, the method developed by Marquez et al.^31^ was employed, in which Fe ions are adsorbed onto the surface of insoluble Ni(OH)_2_. It is evident in Figure 1 that the presence or excess of iron did not impact the onset potential or the current at the lower overpotentials. However, it did have a slight impact at the higher current densities, particularly above 50 mA cm^–2^. Consequently, the CoNiFe-LDH/G composites were studied and statistically optimized at 10 mA cm^–2^ in the commercial KOH solution as the purification process is costly and time-consuming for large-scale operations due to the numerous steps and chemicals involved.

### 3.2 Selection of the Co_[m]_Ni_[3]_Fe_[3]_-LDH/G_[n]_ Composite

**Figure 2:**
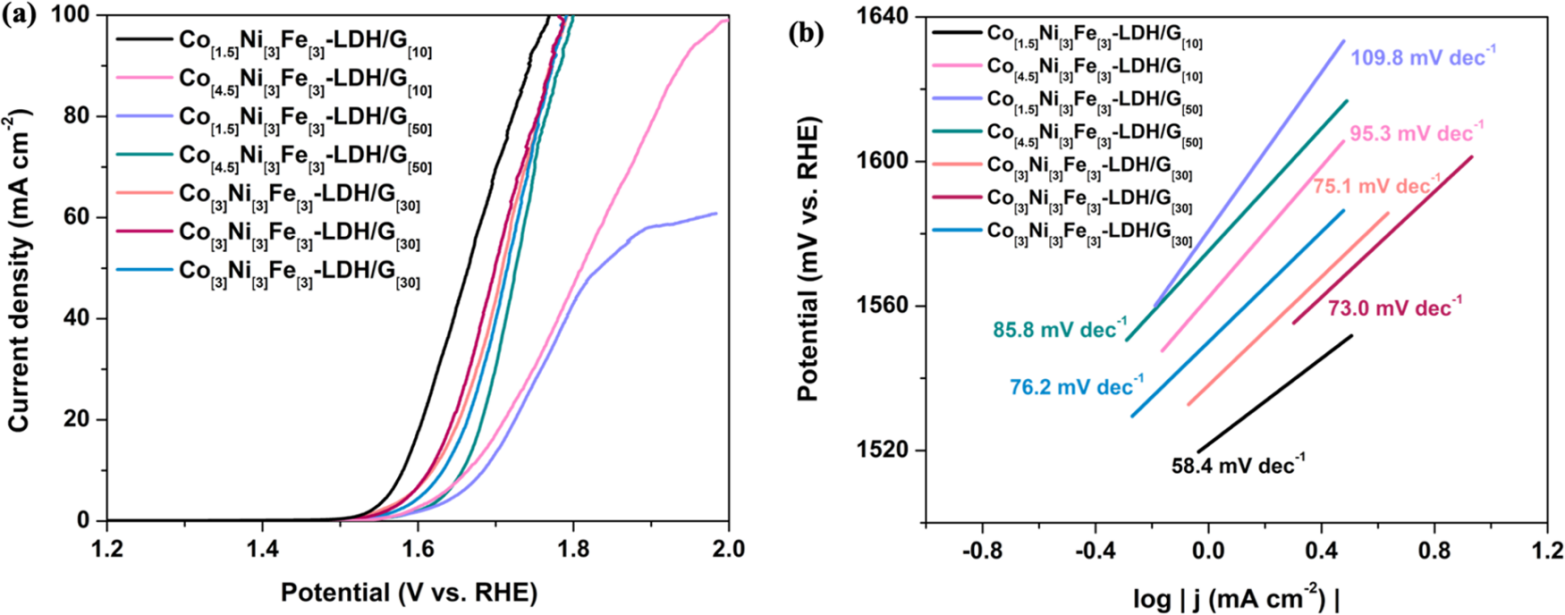
(a) The OER LSV curves of the composites synthesized with different molar ratios of Co(NO_3_)_2_ and amounts of graphene. (b) Tafel slopes derived from the LSV curves.

Factorial design is a useful approach for studying how different parameters affect the optimization of a specific process. In the context of a full-factorial experimental design, measurements of responses are conducted across the complete array of combinations formed by varying the levels of the experimental parameters. For this study, a 2-level full-factorial design was used to evaluate the influence of cobalt and graphene concentrations (independent parameters) on the performance of the NiFe-LDH. The potential equivalent to a current density of 10 mA cm^–2^ during the OER (dependent variable), was the chosen response parameter. Five combinations of dependent variables were used in the optimization experiments. The polarization curves and Tafel slopes of the Co_[m]_Ni_[3]_Fe_[3]_-LDH/G_[n]_ composites for OER are shown in Figure 2a,b, respectively. Additionally, the OER activity was studied in triplicate using the Co_[3]_Ni_[3]_Fe_[3]_-LDH/G_[30]_ (central point) to estimate the variability, which was determined at 0.58%, indicating a low variability.

As shown in Figure 2a,b, the Co_[1.5]_Ni_[3]_Fe_[3]_-LDH/G_[10]_ provides the best OER activity when compared with the other composites that have higher Co and G concentration levels. An impressive onset potential of 1.54 V, combined with a potential of 1.58 V to generate 10 mA cm^–2^, and a Tafel slope of 58.4 mV dec^–1^ were achieved for the Co_[1.5]_Ni_[3]_Fe_[3]_-LDH/G_[10]_. This highlights the important roles of both cobalt and graphene when designing the optimum electrocatalyst. Interestingly, lower concentrations of Co are preferable, and this may be connected with the development of Co-containing particles with the higher Co contents.^32^ While the presence of graphene may be beneficial with the carbon providing sites for adsorption of the OH^–^ ions,^33^ an excess concentration may lead to agglomeration of the graphene sheets giving poorer dispersion of the transition metal centers, decreasing the number of active sites for catalysis.^34^

To assess the reproducibility of the OER performance using the synthesized composites, all experiments were carried out in triplicate. The corresponding standard deviations (<5%) are reported in Table 3, indicating a high level of experimental consistency and reliability. To determine the impact of the main factors on the OER, the independent and dependent parameters were fitted to the linear model equation. ANOVA analysis was conducted at a 95% confidence level, and the corresponding results are presented in Table 4.

**Table 3:** Potential at 10 mA cm^–2^ with RSD for the Synthesized Electrocatalysts

catalyst	potential at 10 mA cm^–2^ (V)^a^	RSD (%)
Co_[1.5]_Ni_[3]_Fe_[3]_-LDH/G_[10]_	1.58 ± 0.02	2.11
Co_[4.5]_Ni_[3]_Fe_[3]_-LDH/G_[10]_	1.67 ± 0.01	1.42
Co_[1.5]_Ni_[3]_Fe_[3]_-LDH/G_[50]_	1.69 ± 0.01	1.53
Co_[4.5]_Ni_[3]_Fe_[3]_-LDH/G_[50]_	1.67 ± 0.02	2.34
Co_[3]_Ni_[3]_Fe_[3]_-LDH/G_[30]_	1.62 ± 0.01	1.36

a Mean ± standard deviation (*n* = 3).

**Table 4:** A Summary of the ANOVA Tests with the Significance of the Variables

source		*F*-value	*p* < 0.05	remarks
model		17.4	0.005	significant
[Co]		9.5	0.009	significant
[G]		18.7	0.004	significant
[Co][G]		25.0	0.003	significant
model S	0.01			
*R*^2^	0.97			
adjusted *R*^2^	0.92			

**Figure 3:**
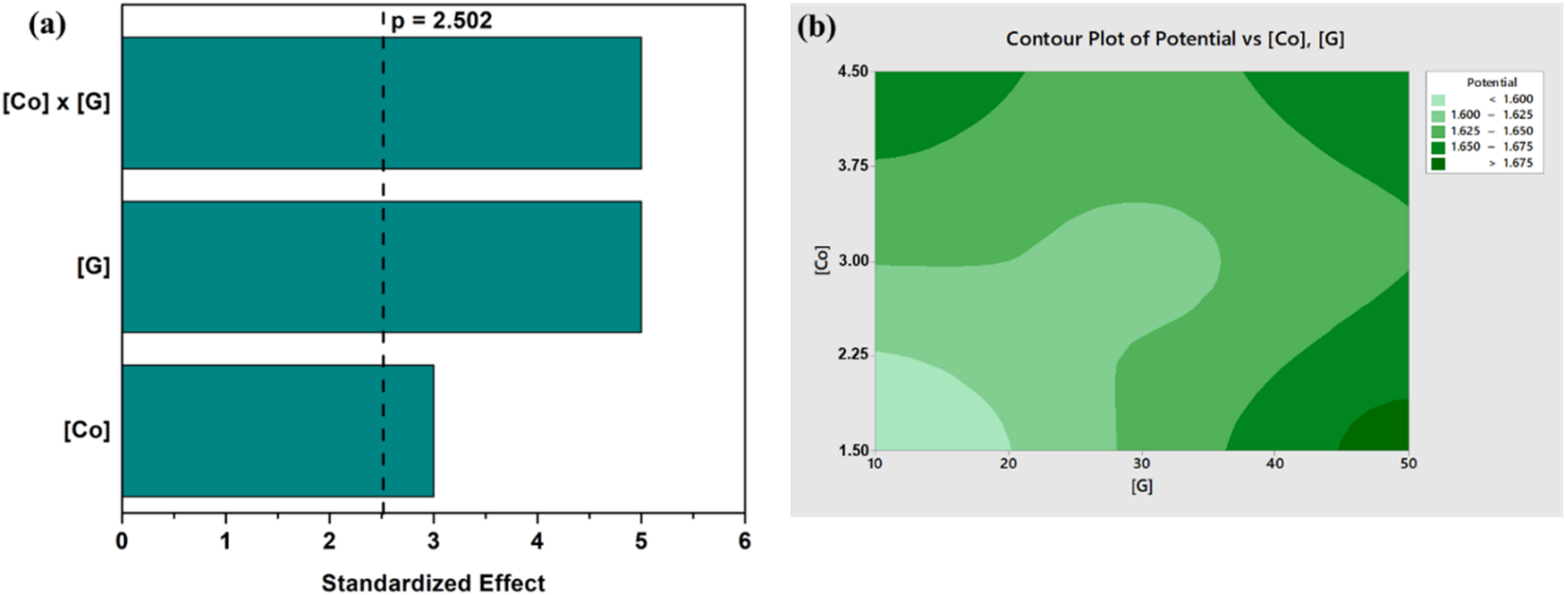
(a) Pareto plot of the standardized effects of 2^2^ full-factorial design (response is potential at 10 mA cm^–2^; α = 0.05); and (b) 2D contour plot of potential at 10 mA cm^–2^ as a function of cobalt concentration and amount of graphene.

A satisfactory prediction regression model, with high determination coefficient values of *R*^2^ = 97.2%, and *R*^2^(adj) = 91.6%, was obtained, confirming that the ANOVA test is validated. The *p-*values are less than 0.05, indicating that the parameters and their interactions are significant, while the *F*-values confirm that the model is significant (*p* < 0.05). In particular, the model terms indicate that the cobalt and graphene concentrations and their interactions, are significant, implying that the OER activity is correlated to these parameters. It was also confirmed by the Pareto plot of effects, illustrated in Figure 3a, that the main effects, cobalt concentration and amount of graphene, and their interaction are significant at a 5% significance level. The response data were fitted to a linear regression equation, according to eq 1, where the potential is expressed in units of V vs RHE and corresponds to the potential required to deliver 10 mA cm^–2^ of current density.
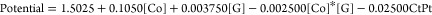
1Here, CtPt corresponds to the central point, [Co] indicates the concentration of cobalt, [G] gives the graphene amounts, and the term [Co]*[G] represents the level of interaction between Co and G. Based on eq 1, it can be confirmed that there is a linear correlation between the factors and the response, with the potential increasing as the cobalt concentration and mass of graphene increases, making the electrocatalysts less favorable for the OER. On the other hand, the interactions between the cobalt and graphene contribute to lowering the potential. This indicates that lower concentrations of Co and graphene are more favorable. Indeed, this can be seen in the 2D contour plot, in Figure 3b, which shows the main and interaction effects of the dependent variables on the potential at 10 mA cm^–2^. This confirms the relationship between the cobalt concentration and the amount of graphene, with the optimum combinations with Co concentrations between 1.5 and 2.25 mM and G between 10 and 20 mg. Therefore, the Co_[1.5]_Ni_[3]_Fe_[3]_-LDH/G_[10]_ was selected as the optimized material for further investigations. To elucidate the contribution of each element to the OER activity, control materials were synthesized and analyzed systematically.

### 3.3 Characterization of the Optimized LDH Composite

In addition to the characterization of the LDH composite, the incorporation of graphene into the LDH was investigated. A combination of XPS, FE-SEM, XRD, and FTIR analyses was employed in the characterization studies, and to elucidate the structural and morphological changes induced by the addition of graphene.

**Figure 4:**
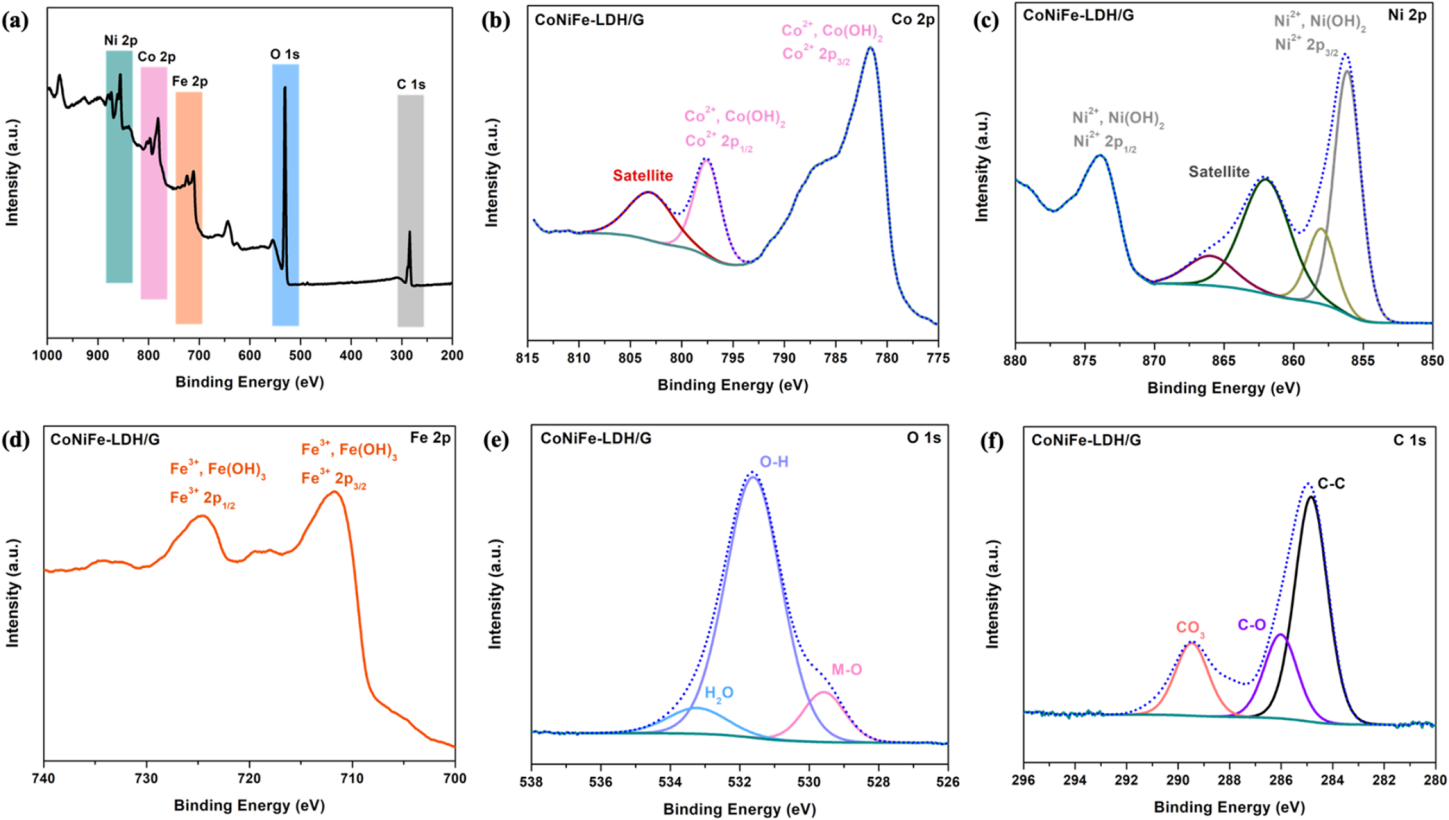
XPS of Co_[1.5]_Ni_[3]_Fe_[3]_-LDH/G_[10]_ (a) Survey, (b) Co 2p, (c) Ni 2p, (d) Fe 2p, (e) O 1s, and (f) C 1s.

The chemical composition, and valence states of the Co_[1.5]_Ni_[3]_Fe_[3]_-LDH/G_[10]_ are summarized in the XPS spectra shown in Figure 4. The XPS survey spectrum, Figure 4a, confirms the presence of Fe, Ni, Co, O and C. The Co 2p spectra in Figure 4b, display two peaks at 781.6 eV (Co^2+^ 2p_3/2_) and 797.5 eV, (Co^2+^ 2p_1/2_), along with a satellite peak at 803.0 eV, confirming the presence of the Co^2+^ species.^35^ The Ni 2p spectra in Figure 4c show characteristic peaks at 856.1 and 874.3 eV, corresponding to the Ni^2+^ 2p_3/2_ and Ni^2+^ 2p_1/2_ energy states, respectively. This is consistent with the Ni^2+^ species within Ni(OH)_2_.^36^ Additional satellite peaks are seen at 862.0 and 866.0 eV, and these suggest the formation of NiO^37^ at the surface. In Figure 4d, two peaks at 711.0 and 724.9 eV corresponding to Fe^3+^ 2p_3/2_ and Fe^3+^ 2p_1/2_, are evident, and this is consistent with previous studies.^38,39^

In Figure 4e, the O 1s spectrum reveals two peaks at 529.6 and 531.6 eV, corresponding to M-O and O–H, respectively. These peaks suggest the formation of hydroxyl interlayer ions in the trimetallic LDHs;^40^ while the peak at 533.2 eV is associated with adsorbed water.^41^ In the C 1s spectrum shown in Figure 4f, a peak at 284.5 eV is identified with C = C and C–C bonds, while another peak at 286.0 eV corresponds to C–O, confirming the successful incorporation of graphene.^42^ Furthermore, a peak at 290.8 eV in the C 1s spectra, corresponds to CO_3_, which may originate from reaction byproducts,^43^ or indicate the presence of intercalated carbonate anions. Collectively, the peaks observed in the spectra for these constituent elements provide evidence for the successful synthesis of the intended trimetallic LDH composites, forming a heterogeneous architecture. Additionally, XPS analysis was performed on the Co_[1.5]_Ni_[3]_Fe_[3]_-LDH composite (Figure S1) to assess the chemical interactions between the LDH and graphene. Notably, a slight narrowing of the peak at 531.6 eV related to M-O bonds upon graphene addition is observed on comparing Figures 4e and S1d. The slight narrowing, around 1/4 of the M-O peak width, in the XPS spectra of graphene-incorporated LDHs can be attributed to electronic interactions between graphene’s π-electron system and the LDH structure, causing subtle charge redistribution around the metal centers and altering their local electronic environment.^44^ Furthermore, the incorporation of graphene can enhance structural organization by reducing defects and improving uniformity, resulting in a more homogeneous chemical environment.^25^ In contrast, the XPS spectra of metal hydroxides showed no noticeable changes, likely due to their stable structure, which prevents significant interaction with graphene. This lack of interaction results in no discernible alterations in their XPS profiles.^45^ The combined effects observed in the LDH-graphene composite, such as the narrowing of the M-O peak, indicate structural modifications that suggest the successful incorporation of graphene into LDHs. This structural enhancement contributes to improved stability and performance of the composite.

**Figure 5:**
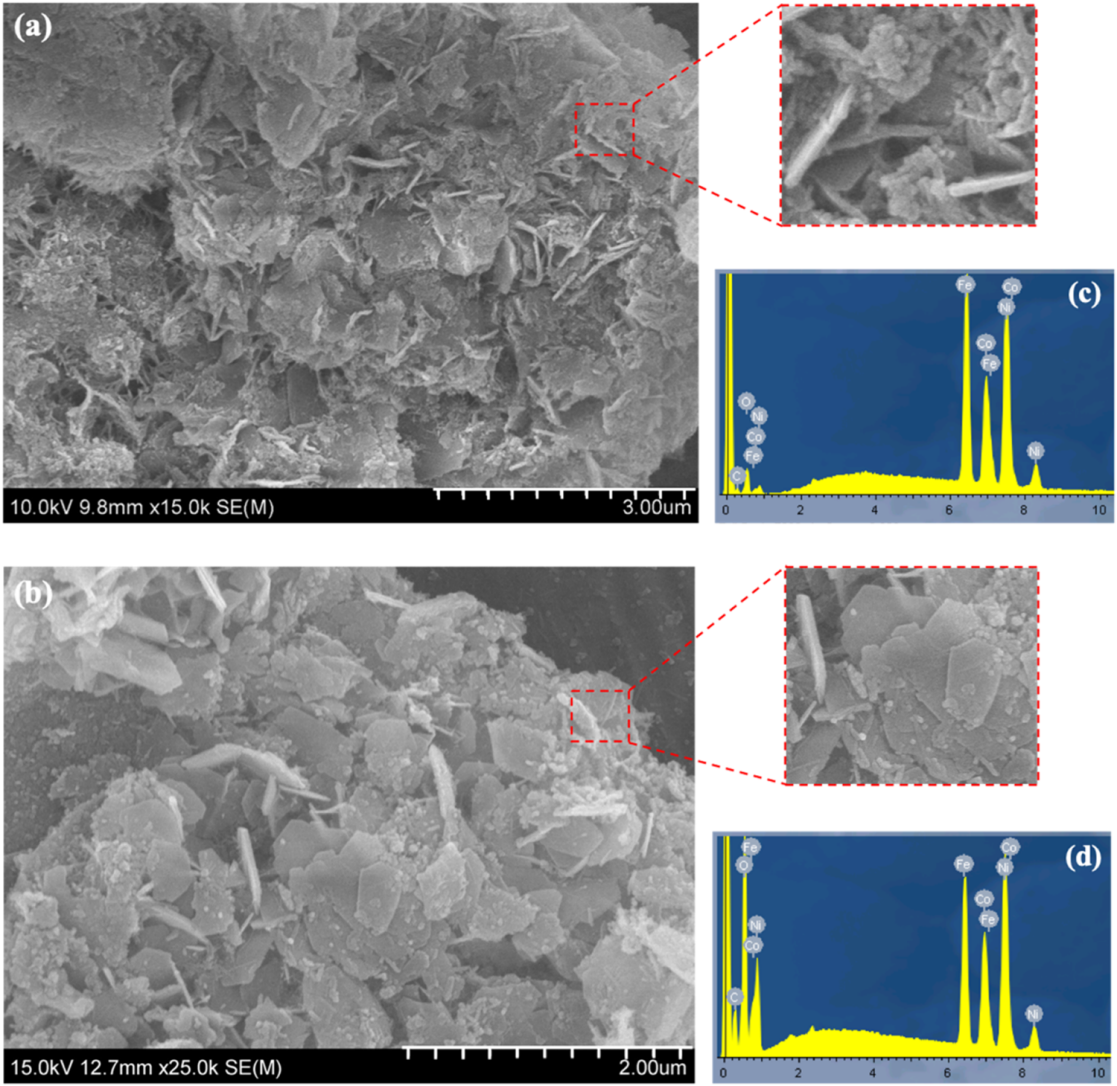
FE-SEM images of (a) CoNiFe-LDH and (b) Co_[1.5]_Ni_[3]_Fe_[3]_-LDH/G_[10]_ EDX spectra for (c) CoNiFe-LDH and (d) Co_[1.5]_Ni_[3]_Fe_[3]_-LDH/G_[10]_.

The morphological changes induced by graphene incorporation were further examined using FE-SEM, as shown in Figure 5. The surface of CoNiFe-LDH, Figure 5a, exhibits a characteristic two-dimensional flake structure. The compact CoNiFe-LDH flakes intersect, giving rise to the formation of clusters, and exhibit a vertical arrangement, in accordance with recent experimental observations.^46^ Furthermore, the CoNiFe-LDH nanosheets are stacked layer by layer, exhibiting a compact arrangement, which results in a dense surface coating. The morphology modification of the trimetallic surface with the insertion of graphene can be observed in Figure 5b. The metal layer of CoNiFe-LDH is positively charged, while the graphene nanoplates are negatively charged.^47^ Hence, electrostatic attraction facilitates the adsorption of graphene onto the CoNiFe-LDH surface, leading to the formation of the LDH-G coating with graphene nanoplatelets covering its surface – a characteristic structure of graphene.^35,48^ Furthermore, examination of the sectional FE-SEM image of CoNiFe-LDH and Co_[1.5]_Ni_[3]_Fe_[3]_-LDH/G_[10]_ reveals a close attachment and coverage of graphene on the surface of Co_[1.5]_Ni_[3]_Fe_[3]_-LDH/G_[10]_. This is consistent with the data in Table 1, where the *p-*values indicate a significant interaction between the Co and G.

The EDX spectra, in Figure 5c,d, suggest that the main elements on both LDH surfaces are Co, Ni, Fe and O, confirming the successful development of the CoNiFe-LDH. However, the Co_[1.5]_Ni_[3]_Fe_[3]_-LDH/G_[10]_, Figure 5d, exhibits a higher relative carbon and oxygen concentration, compared to CoNiFe-LDH (Figure 5c). This is associated with the insertion of graphene sheets, which are decorated with oxygen-containing functional groups, such as C–O groups and oxygen vacancies,^49^ into the LDH which is beneficial in the transfer of electrons from the LDH to graphene.^50^

**Figure 6:**
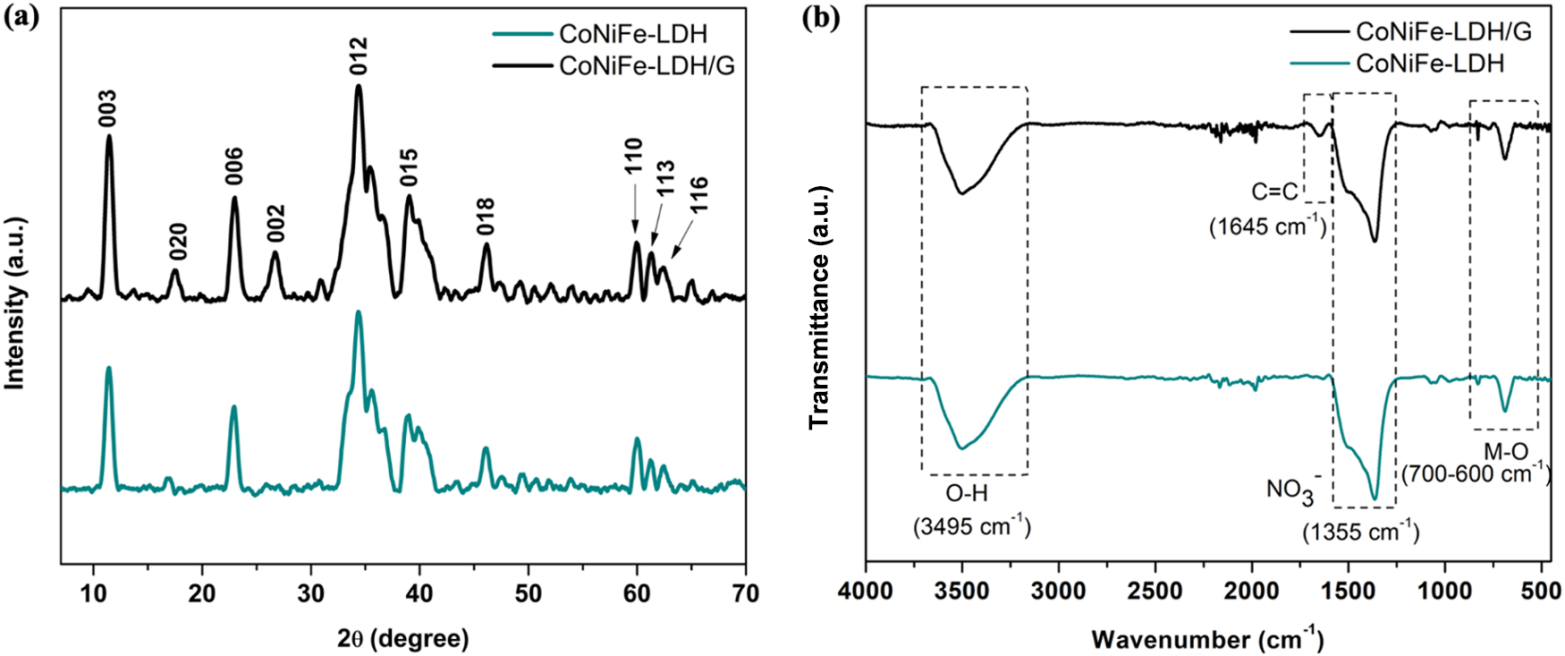
(a) XRD and (b) FTIR spectra of CoNiFe-LDH and Co_[1.5]_Ni_[3]_Fe_[3]_-LDH/G_[10]_.

Crystallographic analysis via XRD provided further insights into the structural integration of graphene with the LDH, as presented in Figure 6a. The XRD pattern of CoNiFe-LDH shows diffraction peaks at 2θ = 11.5, 17.6, 23.4, 34.4, 38.9, 46.2, 59.9, 61.5, and 62.6° which can be indexed to the (003), (020), (006), (012), (015), (018), (110), (113), and (116) planes of the LDH phase.^17,51^ This is a clear indication of the successful synthesis of CoNiFe-LDH. The XRD of the Co_[1.5]_Ni_[3]_Fe_[3]_-LDH/G_[10]_ shows a similar diffraction pattern. However, an additional weak peak at 27.26° is seen, corresponding to the (002) plane of graphene.^48,52^ This confirms the successful formation of Co_[1.5]_Ni_[3]_Fe_[3]_-LDH/G_[10]_.

FTIR spectroscopy was used to obtain further chemical insight into the Co_[1.5]_Ni_[3]_Fe_[3]_-LDH/G_[10]_ and the corresponding graphene-free CoNiFe-LDH. The FTIR spectra were recorded between 4000 and 500 cm^–1^, and typical plots are illustrated in Figure 6b. A broad absorption band is evident at 3495 cm^–1^, and this corresponds to the O–H stretching vibrations of the interlayer H_2_O molecules and is typical of LDHs.^53^ The vibration mode of the NO_3_^–^ ions in the interlayer is evident at 1355 cm^–1^.^54^ The bands observed from 600–700 cm^–1^ are attributed to M–O, M–O–M and O–M–O vibrations where M corresponds to Co, Ni and Fe.^55,56^ Compared with CoNiFe-LDH, the FT-IR spectrum of the Co_[1.5]_Ni_[3]_Fe_[3]_-LDH/G_[10]_ shows an extra weak absorption band at 1645 cm^–1^ which is related to the vibration of C = C at graphene,^57,58^ and this confirms that the graphene nanoplatelets were successfully incorporated throughout the LDH structure.

Overall, the combined XPS, FE-SEM, XRD and FTIR analyses demonstrate that graphene incorporation into the Co_[1.5]_Ni_[3]_Fe_[3]_-LDH structure is facilitated by electrostatic attractions and chemical interactions between functional groups, resulting in enhanced structural stability and potentially improved OER activity.

### 3.4 Electrocatalyst Performance

**Figure 7:**
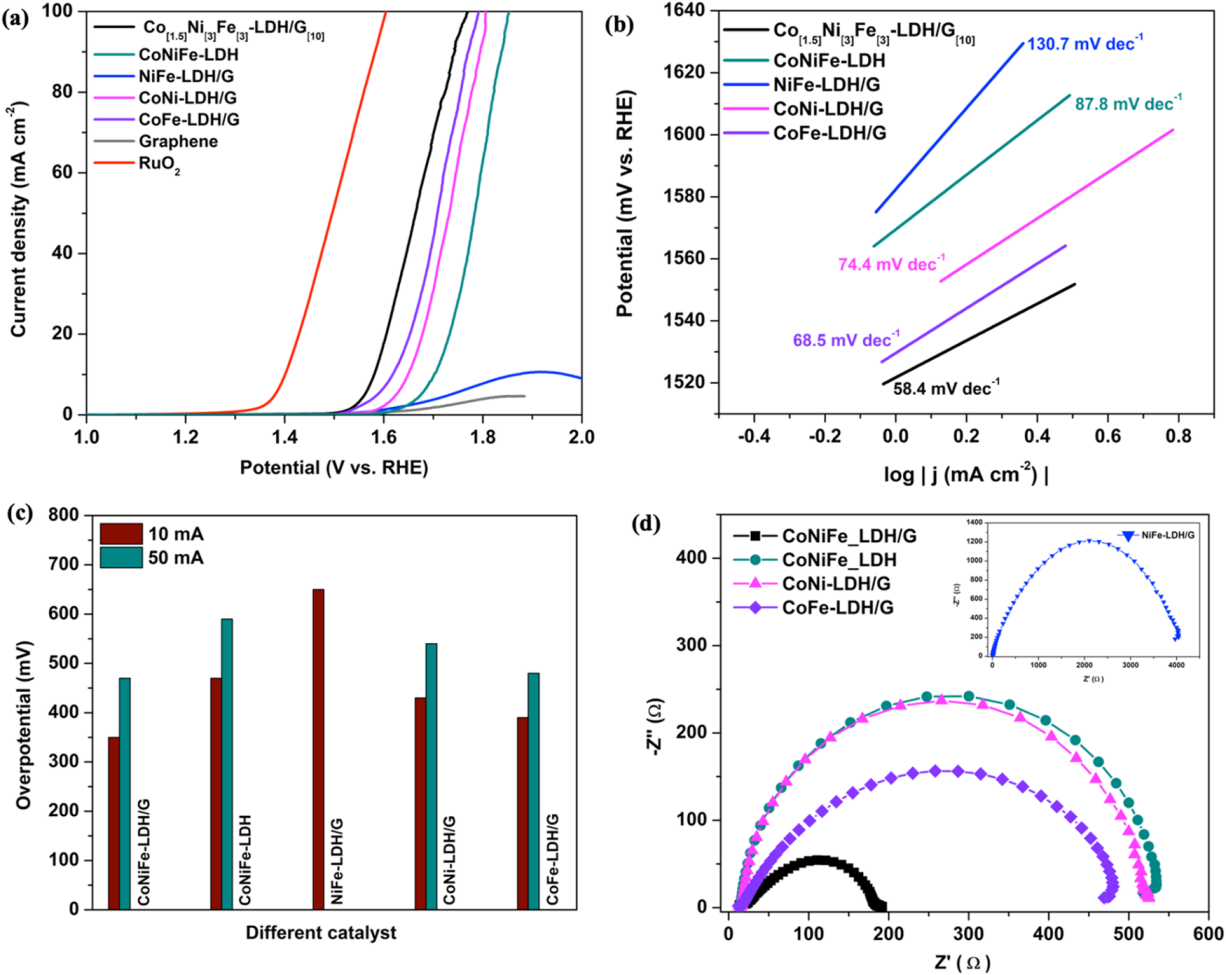
(a) The OER LSV curves, (b) Tafel slopes derived from the LSV curves, (c) histogram of overpotentials at 10 and 50 mA cm^–2^, and (d) electrochemical impedance plots recorded in 1 M KOH.

The OER activity of the Co_[1.5]_Ni_[3]_Fe_[3]_-LDH/G_[10]_, and the two-component control samples, NiFe-LDH/G, CoNi-LDH/G and CoFe-LDH/G were studied and compared to the RuO_2_ benchmark catalyst. The resulting LSV curves are presented in Figure 7a. While RuO_2_ demonstrates a lower onset potential of 1.38 V, the optimized Co_[4.5]_Cu_[3]_Fe_[3]_-LDH/G_[10]_ catalyst shows a comparable onset potential and similar slope, suggesting promising electrochemical behavior. The onset potential and overpotential at 10 mA cm^–2^ (Figure 7c) were selected as the basis for characterizing the catalytic performance of the materials, and these are summarized in Table 5. It can be observed that the optimized Co_[1.5]_Ni_[3]_Fe_[3]_-LDH/G_[10]_ shows the lowest onset potential at 1.54 V corresponding to an overpotential of 350 mV. The NiFe-LDH/G demonstrates the highest onset potential at 1.67 V with an overpotential of 650 mV, highlighting the significant role of cobalt in enhancing the OER performance. This can be attributed to the oxidation of Ni^2+^ to Ni^3+^ promoted by the Co charge transfer effect which in turn gives the higher conductivity NiOOH phase.^59^ Additionally, NiOOH activates the Fe sites which are inaccessible to electron transfer in the Ni(OH)_2_ phase.^17,60^ Meanwhile, the Fe^3+^ ions promote the oxidation of Co^2+^ to Co^3+^, forming CoOOH which further improves the OER activity for the Co_[1.5]_Ni_[3]_Fe_[3]_-LDH/G_[10]_ composite.^61^ Furthermore, CoNiFe-LDH shows a higher onset potential at 1.64 V with an overpotential of 470 mV compared to Co_[1.5]_Ni_[3]_Fe_[3]_-LDH/G_[10]_, suggesting that the added graphene ensures an adequate electron supply during the electrocatalytic process, thereby boosting the OER performance.^62^

**Table 5:** Onset Potential and Overpotential for the Different Composites

composites	onset potential (V)	overpotential (mV)
Co_[1.5]_Ni_[3]_Fe_[3]_-LDH/G_[10]_	1.54	350
CoNiFe-LDH	1.64	470
NiFe-LDH/G	1.67	650
CoNi-LDH/G	1.63	430
CoFe-LDH/G	1.58	390

A comparative analysis with studies from the recent literature, as shown in Table 6, demonstrates that the Co_[1.5]_Ni_[3]_Fe_[3]_-LDH/G_[10]_ exhibits a lower overpotential at 10 mA cm^–2^. This suggests that its electrocatalytic performance is either superior or at least comparable to other reported electrocatalysts for OER. This improved performance can be attributed to the in situ growth of CoNiFe-LDH on the graphene substrate, which effectively mitigates the restacking of graphene sheets and prevents the aggregation of LDH particles. This structural integration fosters strong interactions and synergistic effects between the CoNiFe-LDH and graphene, facilitating enhanced electron transfer and consequently leading to superior electrocatalytic efficiency.^63^

**Table 6:** Performance Comparison of the Optimized LDH with the Literature

composites	KOH electrolyte (M)	overpotential at 10 mA cm^-2^ (mV)	ref.
CoAl-LDH/NG	1.0	365	(64)
N-NiZnCu- LDH/rGO	1.0	489	(42)
CoNiMn- LDH/PPy/rGO	0.1	369	(65)
CoAl-LDH	1.0	415	(64)
NiCe-LDH/CNT	1.0	417	(66)
ZnCo-LDH/rGO	0.1	450	(67)
NiMn-LDH	1.0	520	(68)
CO_3_–CoAl-LDH	1.0	422	(69)
benzoate-Co-LDH	1.0	360	(70)
Mo-NiFe-LDH	1.0	491	(71)
ZnCo-LDH	1.0	420	(72)
Co_[1.5]_Ni_[3]_Fe_[3]_-LDH/G_[10]_	1.0	350	this work

Additional insights into the kinetics and mechanism of the OER were obtained using Tafel analysis. The Tafel equation is described in eq 2, where the slope (*b*) determines how rapidly the current density (*i*) increases with an increase in overpotential (η), while the constant (*a*) depends on the exchange current density. The LSV curves are presented in Figure 6a, while the Tafel regions and the associated slopes are shown in Figure 6b.
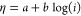
2

A low Tafel slope of 58.4 mV dec^–1^ was obtained for the Co_[1.5]_Ni_[3]_Fe_[3]_-LDH/G_[10]_, Figure 7b. This combined with the low overpotential relative to the other LDHs, Table 4, indicates that this optimized system has an electronic structure that permits more efficient adsorption/desorption of the oxygenated species, giving rise to a higher OER activity.^73^ Additionally, the CoNiFe-LDH without graphene has a much higher Tafel slope of approximately 94.5 mV dec^–1^, clearly highlighting the significant role of graphene in the Co_[1.5]_Ni_[3]_Fe_[3]_-LDH/G_[10]_ composite.

Further information on the electronic conductivity of the composites was obtained using electrochemical impedance spectroscopy (EIS). The equivalent circuits employed for fitting the impedance data are shown in Figure S2 and involve the solution resistance, (*R*_s_), charge transfer resistance (*R*_CT_) and a constant phase element (CPE). The impedance of the Co_[1.5]_Ni_[3]_Fe_[3]_-LDH/G_[10]_ composite can be fitted to a simple Randles cell, while an additional constant phase element is required with the NiFe-LDH/G. The plots are characterized by a depressed semicircle, Figure 7d. The diameter of the semicircle, which corresponds to the *R*_CT_, varies considerably depending on the nature of the LDHs. Cearly the Co_[1.5]_Ni_[3]_Fe_[3]_-LDH/G_[10]_ composite has the lowest charge transfer resistance.

The *R*_CT_ values for the various LDH composites are compared in Table 7. Clearly, the Co_[1.5]_Ni_[3]_Fe_[3]_-LDH/G_[10]_ exhibits the lowest R_CT_, at 188.1 ± 4.58 Ω, and this is consistent with its lower onset potential, lower overpotential and favorable Tafel slope. Indeed, the *R*_CT_ obtained for Co_[1.5]_Ni_[3]_Fe_[3]_-LDH/G_[10]_ is around 20 times lower compared to NiFe-LDH/G, which has the highest *R*_CT_ of 3673.0 ± 7.12 Ω. Furthermore, the CoNiFe-LDH without graphene has a higher *R*_CT_ of 529.30 ± 4.58 Ω. Consequently, the incorporation of G and Co into the Co_[1.5]_Ni_[3]_Fe_[3]_-LDH/G_[10]_ improves its electronic conductivity, expediting the charge transfer rate in the OER process.^74^

**Table 7:** Electrochemical Impedance Parameters

composites	*R*_CT_ (Ω)	*R*_CT2_ (Ω)
Co_[1.5]_Ni_[3]_Fe_[3]_-LDH/G_[10]_	188.10 ± 4.58	
CoNiFe-LDH	529.30 ± 4.58	
NiFe-LDH/G	354.70 ± 6.20	3673.0 ± 7.12
CoNi-LDH/G	511.50 ± 5.24	
CoFe-LDH/G	503.90 ± 5.83	

**Figure 8:**
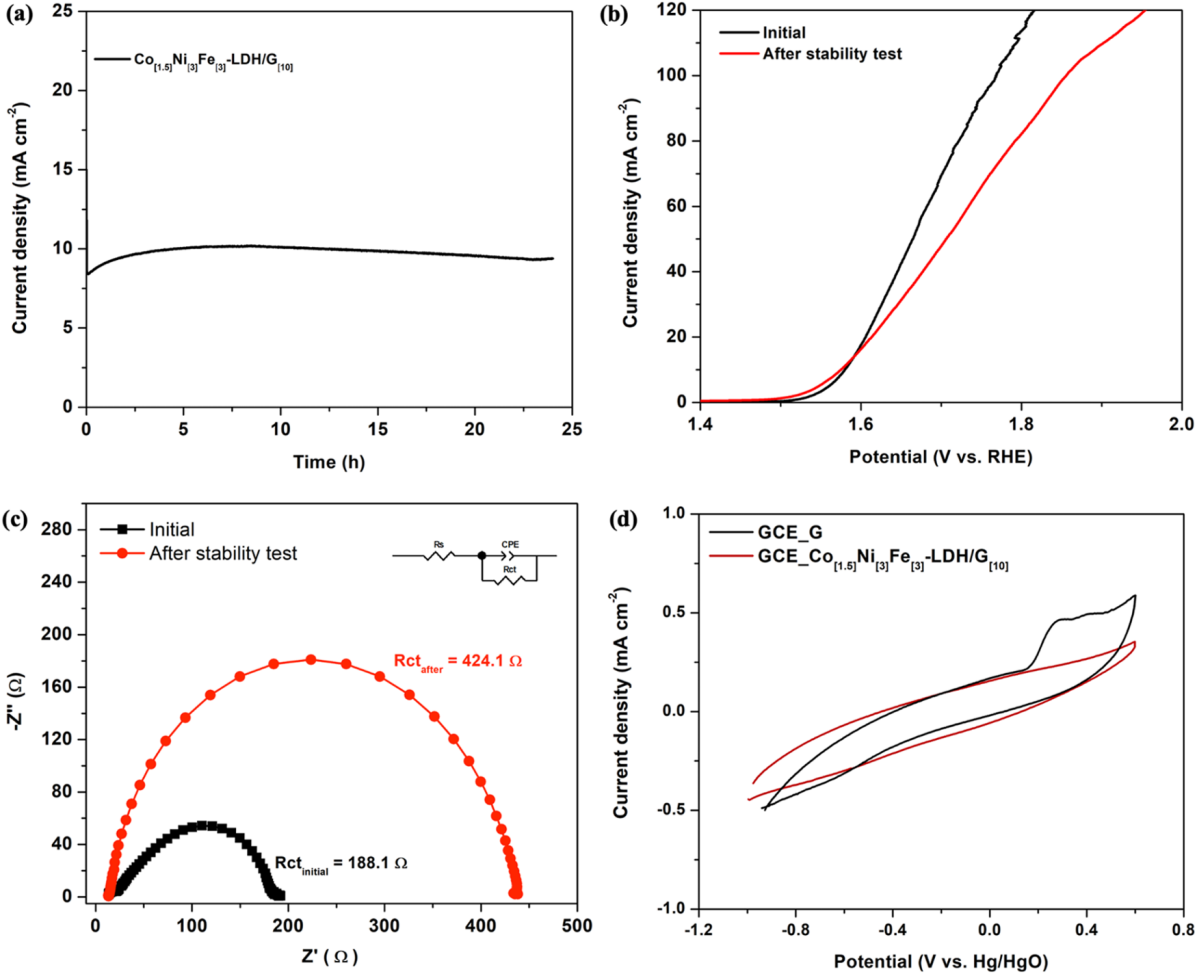
(a) A 24 h-stability test of Co_[1.5]_Ni_[3]_Fe_[3]_-LDH/G_[10]_ at 10 mA cm^–2^, (b) polarization curves before and after stability test, (c) electrochemical impedance plot before and after stability test, and (d) CV in a buffer (pH 7.0) for graphene dispersed onto GCE and Co_[1.5]_Ni_[3]_Fe_[3]_-LDH/G_[10]_ after 24 h at 10 mA cm^–2^.

It is widely known that stability is a critical factor in determining whether electrocatalysts can be practically applied.^75^ Therefore, the stability of the Co_[1.5]_Ni_[3]_Fe_[3]_-LDH/G_[10]_ was examined using chronoamperometry as a fixed potential of 1.58 V (RHE) equivalent to 10 mA cm^–2^. The corresponding current–time plot is illustrated in Figure 8a. The current density decreases slightly to 9.48 mA cm^–2^ after 24 h due to the accumulation of bubbles on the active sites.^24^ This indicates that there is only a negligible degradation in current density after a 24-h period, confirming that the Co_[1.5]_Ni_[3]_Fe_[3]_-LDH/G_[10]_ has advantageous durability and catalytic stability.

The stability was further evaluated using LSV curves, Figure 8b, and electrochemical impedance spectroscopy, Figure 8c, indicating differences before and after the stability test. While the onset potential is slightly improved after the 24 h period, there are more significant changes in the higher current densities. Additionally, the charge transfer resistance (*R*_CT_) increases after the stability test from 188.1 to 424.1 Ω. The polarization of the catalyst at these high anodic potentials for the long-term of 24 h may cause its oxidation leading to some degradation of the Co_[1.5]_Ni_[3]_Fe_[3]_-LDH/G_[10]_ electronic conductivity.^75^

It is clear from Figure 7 that the graphene sheets facilitate the OER. However, in an alkaline environment, graphene sheets are susceptible to oxidation, especially at the potentials required for OER. To assess this, the stability of the graphene was compared with the graphene immobilized within the Co_[1.5]_Ni_[3]_Fe_[3]_-LDH/G_[10]_. Following a 24-h stability period at 1.58 V, cyclic voltammograms were recorded in a neutral phosphate buffer. The CV curves showed consistent behavior over 30 cycles, and the final cycles are plotted in Figure 8d. The two profiles exhibit distinct differences, particularly with the appearance of a peak at 0.4 V, indicative of graphene oxidation.^48^ The absence of this peak at 0.4 V in the Co_[1.5]_Ni_[3]_Fe_[3]_-LDH/G_[10]_ CV profile indicates that the graphene maintained its structural integrity when incorporated into the composite. This enhanced stability is likely to be due to the protective effect of the LDH phase, which appears to effectively prevent oxidation of the graphene for at least 24 h.

It is well established that scaling up electrochemical systems typically requires higher current densities. Therefore, to evaluate the stability of the composite under such conditions over an extended duration, a chronoamperometry experiment was performed at a fixed potential of 1.66 V (RHE), corresponding to a current density of 50 mA cm^–2^, for a period of 72 h. The resulting current–time profile shows an initial decrease in current density over the first 14 h, stabilizing at 44.5 mA cm^–2^, as presented in Figure S3a. Beyond this period, the current density remained constant for the remaining 58 h, indicating that despite an initial decline, the composite maintained stability at 44.5 mA cm^–2^. This stability was further supported by LSV curves recorded before and after the test, Figure S3b, which showed a slight increase in onset potential after 72 h, with more pronounced changes at higher current densities, as seen previously at 10 mA cm^–2^.

Additionally, SEM was utilized to analyze the surface morphology of the composite both before and after the stability test, as shown in Figure S4a,b, respectively. Unlike the powder form of CoNiFe-LDH/G, the composite layer on GE exhibits a more compact morphology (Figure S4a) with minimal changes observed after the stability test (Figure S4b). This suggests that the material retains its morphology even under elevated current densities and prolonged operational conditions.

Furthermore, to evaluate potential metal leaching, the residual KOH solution after the 72 h stability test was analyzed using ICP. The elemental concentrations of Co, Ni, and Fe, expressed in ppb, are summarized in Table 8. The results indicate that the composite exhibits minimal metal leaching over the long-term test, as evidenced by the low concentrations of Co, Ni, and Fe in solution. Notably, Fe was detected at a higher concentration, which is consistent with the initial analysis of the unpurified KOH solution, where Fe impurities are present.

**Table 8:** ICP-MS Analysis of Co, Ni, and Fe Concentrations in 1M KOH Solution Following a 72 h-Stability Test at 50 mA cm^–2^

wavelength (nm)	Co (ppb)^a^	Ni (ppb)^a^	Fe (ppb)^a^
230.79	6.42 ± 0.03		
238.89	5.33 ± 0.02		
216.55		6.82 ± 0.06	
231.60		6.00 ± 0.02	
238.20			15.31 ± 0.33
261.19			9.19 ± 0.30

a Mean ± standard deviation (*n* = 3).

## 4 Conclusions

In this study, Co_[m]_Ni_[3]_Fe_[3]_-LDH/G_[n]_ composites were synthesized through a single-step hydrothermal reaction. The influence of cobalt and graphene concentrations on the OER performance was examined using a 2-level full factorial design to optimize the LDH composite for enhanced OER performance. The Co_[1.5]_Ni_[3]_Fe_[3]_-LDH/G_[10]_ composite reached the lowest onset potential of 1.54 V, the potential at 10 mA cm^–2^ was 1.58 V, and the Tafel slope was 58.4 mV dec^–1^, indicating that a lower concentration of cobalt and graphene has the optimum combination for the OER process. Furthermore, the ANOVA analysis showed that both cobalt and graphene concentrations and their interactions are statistically significant, with a linear correlation between them. Also, the optimized composite showed good electronic properties and stability, without losing significant catalytic activity over a 24-h period. Additionally, the longer stability test at 50 mA cm^–2^ demonstrates a stable performance at 44.5 mA cm^–2^ for 58 h after an initial decline in current density. This stability was further supported by LSV analysis, which showed minor changes in onset potential and more significant changes in higher current density over 72 h. SEM imaging confirmed that the composite retains its compact morphology under prolonged operational conditions, while ICP-MS analysis indicated minimal metal leaching, with a slightly higher Fe concentration primarily attributed to impurities in the KOH solution. These findings highlight the composite’s reliability for electrochemical applications under elevated current densities. Therefore, this study provides a facile and efficient strategy to design and optimize a trimetallic LDH combined with graphene as an electrocatalyst to enhance the OER activity.

## Supporting Information Available

XPS of Co_[1.5]_Ni_[3]_Fe_[3]_-LDH (a) Co 2p, (b) Ni 2p, (c) Fe 2p, and (d) O 1s (Figure S1); equivalent circuits of (a) CoNiFe-LDH/G, CoNiFe-LDH, CoNi-LDH/G, and CoFe-LDH/G; (b) NiFe-LDH/G (Figure S2); (a) 72 h-stability test of Co_[1.5]_Ni_[3]_Fe_[3]_-LDH/G_[10]_ at 50 mA cm^–2^, (b) polarization curves before and after stability test (Figure S3); SEM images of Co_[1.5]_Ni_[3]_Fe_[3]_-LDH/G_[10]_ on the glassy carbon electrode: (a) before the stability test and (b) after the stability test at 50 mA cm^–2^ for 72 h (Figure S4) (PDF)
