# Supplementary material for: Factorial Design and Optimization of Trimetallic CoNiFe-LDH/Graphene Composites for Enhanced Oxygen Evolution Reaction
Source: ACS Appl Energy Mater. 2025 Apr 7;8(8):5455–67. doi: 10.1021/acsaem.5c00483 (PMC12042162; doi:10.1021/acsaem.5c00483)
Supplement: Supplementary file 1 — ae5c00483_si_001.pdf [file ae5c00483_si_001.pdf]

Factorial design and optimisation of trimetallic CoNiFe-LDH/Graphene composites for enhanced oxygen evolution reaction

Daniele Alves[1*], Gillian Collins[1], Marilia B Dalla Benetta[1], Eithne Dempsey[1,2], Jae-Jin Shim[3], Raj Karthik[3], and Carmel B Breslin[1,2]

[1]Department of Chemistry, Maynooth University, Maynooth, Co. Kildare, Ireland, W23 F2H6
[2]Kathleen Lonsdale Institute, Maynooth University, Maynooth, Co, Kildare, Ireland, W23 F2H6
[3]School of Chemical Engineering, Yeungnam University, Gyeongsan, Republic of Korea, KR 38541

*Corresponding author: Daniele Alves (daniele.alves@mu.ie)

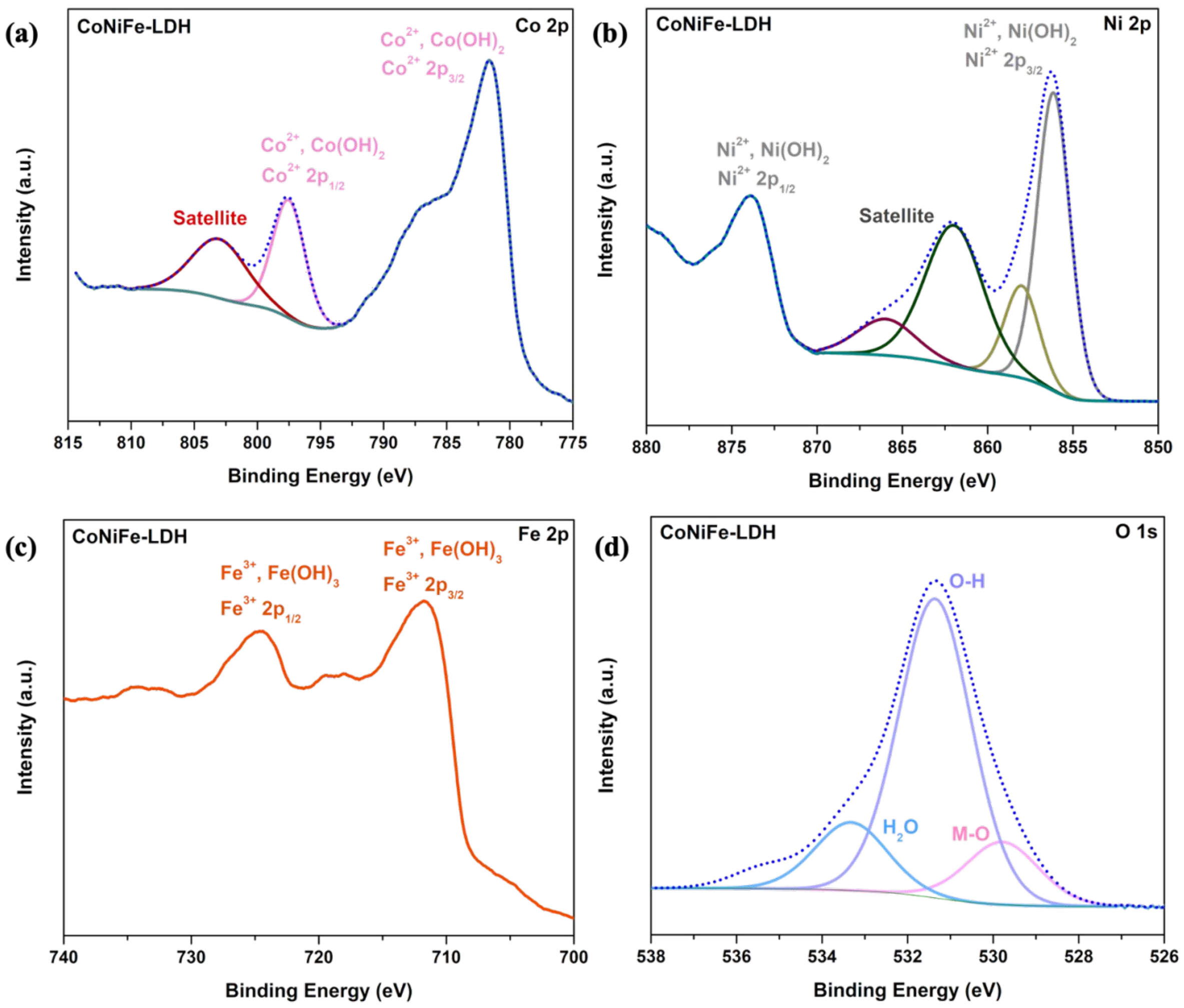

Figure S1. XPS of $Co_{[1.5]}Ni_{[3]}Fe_{[3]}$-LDH (a) Co 2p, (b) Ni 2p, (c) Fe 2p, and (d) O 1s.

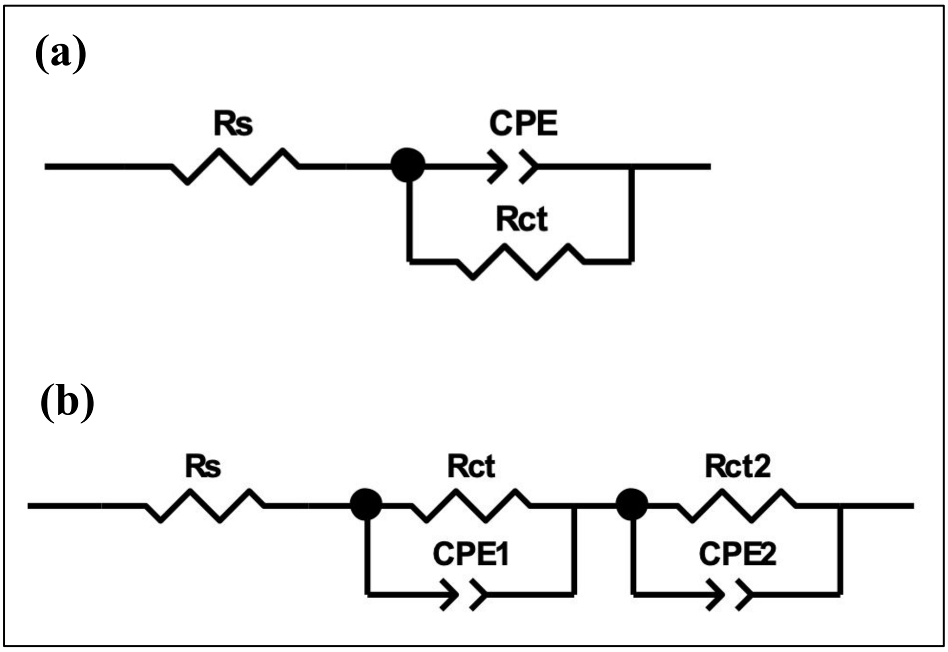

Figure S2. Equivalent circuits of (a) CoNiFe-LDH/G, CoNiFe-LDH, CoNi-LDH/G and CoFe-LDH/G; (b) NiFe-LDH/G.

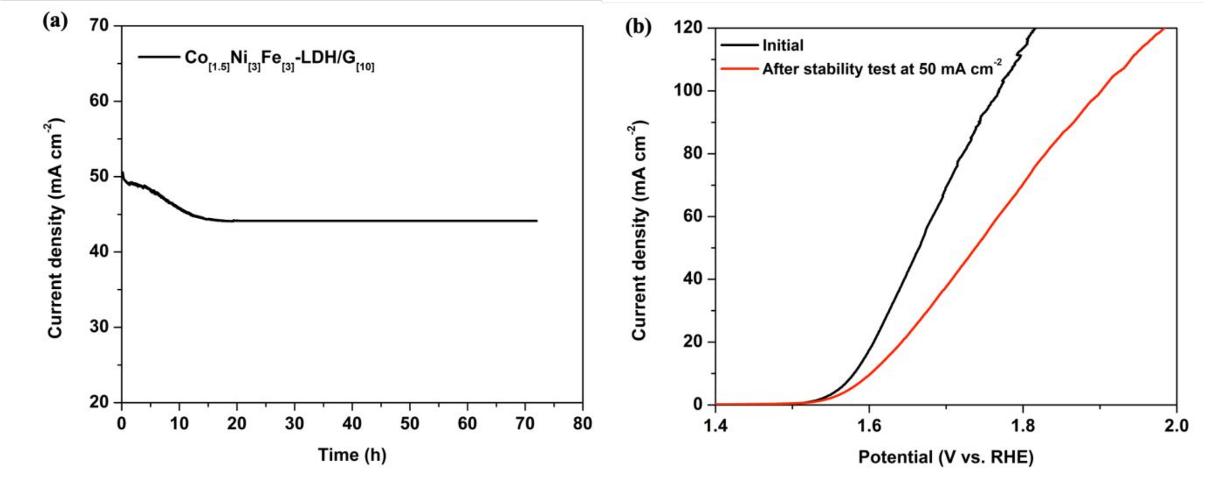

Figure S3. (a) 72 h-stability test of $Co_{[1.5]}Ni_{[3]}Fe_{[3]}$-LDH/$G_{[10]}$ at 50 mA cm^{-2}, (b) Polarisation curves before and after stability test.

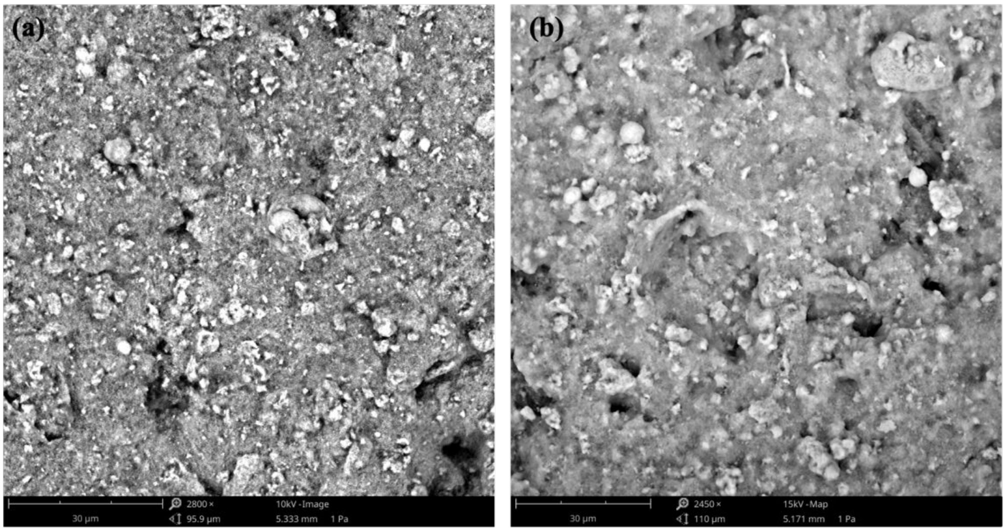

Figure S4. SEM images of $Co_{[1.5]}Ni_{[3]}Fe_{[3]}$-LDH/$G_{[10]}$ on the glassy carbon electrode: (a) before the stability test and (b) after the stability test at 50 mA cm^{-2} for 72h.
